# Real time *in situ* x-ray diffraction study of the crystalline structure modification of Ba_0.5_Sr_0.5_TiO_3_ during the post-annealing

**DOI:** 10.1038/s41598-018-30392-y

**Published:** 2018-08-10

**Authors:** Sondes Bauer, Adriana Rodrigues, Tilo Baumbach

**Affiliations:** 1 Institute for Photon Science and Synchrotron Radiation IPS, Karlsruhe Institute of Technology KIT, Hermann-von-Helmholtz-Platz 1, D-76344, Eggenstein-Leopoldshafen, Germany; 2 Laboratory for Application of Synchrotron Radiation LAS, Kaiserstraße 12 76131, Karlsruhe, Germany

## Abstract

We report about an *in situ* study of crystalline structural changes during thermal treatment of a Ba_0.5_Sr_0.5_TiO_3_ (BSTO) film grown on MgO. The study covers the complete cycle of heating, annealing and cooling and reveals simultaneous phenomena of phase transitions and strain evolution, which have been characterized by *in situ* 2D reciprocal space mapping (2D-RSM) using high-resolution synchrotron x-ray diffraction in coplanar and grazing incidence geometries. In this way, temperature induced phase transformation from the BSTO2 to the BSTO1 phase has been monitored and the appearance of a further crystalline phase was detected. Moreover, for both BSTO phases, transitions between in-plane compressive and tensile states have been determined during thermal treatment. Furthermore, a contraction of the out-of-plane lattice components has been observed during the annealing phase while the in-plane lattice components remain leading to the change of the residual in-plane strain towards tensile state. The *in situ* 2D-RSM findings provide valuable and versatile insights into strain engineering and structure modification upon thermal treatment.

## Introduction

The dielectric properties depend strongly on the microstructure which can be influenced by the growth conditions and by the possible post-growth processing. Strain engineering is a relevant approach to affect significantly the dielectric properties^1–3^. Several processing methods such as film thickness^4^, oxygen pressure^5^ and post-annealing^1,6^ were demonstrated to affect efficiently and remarkably the residual strain in the grown film, and consequently the ferroelectric properties. Furthermore, it should be emphasized that the tunability of BSTO grown on MgO reaches a value higher than *60%* when the film thickness exceeds a critical thickness of *150* *nm* as it could be demonstrated by Bellotti *et al*.^7^. There have been some attempts to understand the connection between the change in the microstructure and the improvement of the dielectric performance of the BSTO films. Carlson *et al*.^1^ have associated the improvement in the dielectric properties to the high structural quality of the as-deposited films while Tse *et al*.^8^ have attributed the increase of *40%* in the dielectric tunability to the reduction of *40%* in the threading dislocation density due to the post annealing of epitaxial Ba_0.5_Sr_0.5_TiO_3_ thin films grown on (001) MgO by pulsed laser deposition.

The effect of post annealing on the crystal structure and dielectric properties of Ba_x_Sr_(1−x)_TiO_3_ films has been studied by several research groups. An improvement in the dielectric behaviour was found by post annealing^1,9–11^. Knauss *et al*.^12^ have demonstrated that the dielectric losses in the annealed films are lower than in the as-deposited films in the high temperature range and the annealed film had greater tunability with reduced loss. This has been achieved through different *ex situ* investigations carried out on grown films in the as deposited state and in the *ex situ* post-annealed state in terms of microwave dielectric properties. The main aim of the post-annealing is the introduction of oxygen atoms in order to eliminate the oxygen vacancies, and to reduce defects and to increase grain size as it has been stated by Sekhar^13^.

A correlation was observed by Horwitz *et al*.^6^ between the dielectric constant, the electric field effect and the stress in the deposited film which depends on the post-annealing process and the post-annealing temperature. Furthermore, Tumarkin *et al*.^14^ have studied the influence of annealing temperature *T*_*an*_ on the chemical and crystalline phase composition on RF magnetron BSTO grown films. By measuring the XRD pattern, they found out that the annealing of BSTO film at *T*_*an*_ = *750* *°C* at a temperature higher than that of the substrate (*T*_*s*_ = *700* °C) may introduce changes in the chemical and phase composition. Additionally, they characterise the dielectric properties of the films annealed at *T*_*an*_ below *T*_*s*_ and they did not detect any variation in the capacitance and the tunability.

The influence of the annealing temperature on the residual strain was studied by Chang *et al*.^15^, who demonstrated that the as-deposited films on MgO substrates are in tension parallel to the film surface. However, the films annealed at a temperature below *1000* *°C* change strain towards compression. This annealing study was restricted to the characterization of the crystalline structure in terms of lattice parameter and in-plane and out-of-plane strain in the as-deposited and in the post-annealed states. The highest dielectric constant and dielectric tunability was obtained in a film with a slight tensile strain where the distortion ratio comprises between *0*.*9992* and *1*.*00023*^16^.

In this context, X-ray diffraction was employed to measure the in-plane and the out-of-plane lattice parameters of Ba_x_Sr_1−x_TiO_3_ determined from the diffraction peaks of the rocking curve with the only goal to determine the mean residual strain in the BSTO grown film^1,5,9,13,14^. Moreover, only a little attention was devoted to the changes in the shape of diffraction profiles and to the analysis of the crystalline structure in terms of crystal quality, size and defects. To our knowledge, there were no *in situ* diffraction studies, either during the annealing process or during the heating and the cooling steps.

Bauer *et al*.^17^ have demonstrated the usefulness of *in situ* 2D-RSM for the strain evolution of Ba_0.5_Sr_0.5_TiO_3_ thin films grown on MgO using real-time synchrotron x-ray diffraction. In this latter study, the authors have demonstrated the diffraction profile becoming asymmetric with the increase of the film thickness, indicating the formation of two phases when the film thickness exceeds a certain thickness. Furthermore, two separated diffraction peaks were distinguished in the 2D-RSM of the diffraction profiles of XRD002 reflections for thick layers beyond *310* *nm*.

In the present work, we report on a detailed *in situ* x-ray diffraction analysis during the heating, annealing, and cooling steps, with the goal to study the evolution of the content of different BSTO phases as well as the related strain behaviour. For this purpose, *in situ* 2D-RSM in coplanar symmetric x-ray diffraction and non-coplanar grazing incidence diffraction geometries have been recorded to measure the Bragg peaks and their profiles and to determine the changes in out-of-plane and in-plane lattice parameters with the aim to conclude on the residual strain and on the tetragonal distortion with temperature. Additionally, the fitting analysis of the radial diffraction profiles of the XRD200 and of GID200 is given as by Figs (2 and 3) as part of the supplementary information. All the derived fitting parameters corresponding to the heating/annealing and cooling phases are also summarised in the supplementary tables as part of the supplementary information.

## Results and Discussion

### Evolution of the crystalline BSTO phases during thermal treatment

We report on the post-growth thermal treatment in oxygen background with a pressure of *450 mTorr* to a Ba_0.5_Sr_0.5_TiO_3_ BSTO film of about *760* *nm* thickness grown on [001] oriented MgO substrates by pulsed laser deposition. We studied the changes in the crystalline structure by *in situ* by XRD during the treatment which was characterized by a heating rate of *150* *°C/min*, an annealing phase of *180* *min* at *T* = *900* *°C* higher than the growth temperature *Tg* = *850* *°C* and a cooling rate of *150* *°C/min* (see also section ***data acquisition procedure***). Furthermore, once the temperature step is reached, the acquisition of RSMs of XRD002 and GID200 take about *15* minutes. This leads to a smoothed heating/cooling rate of only *10* *°C/min*.

**Figure 1:**
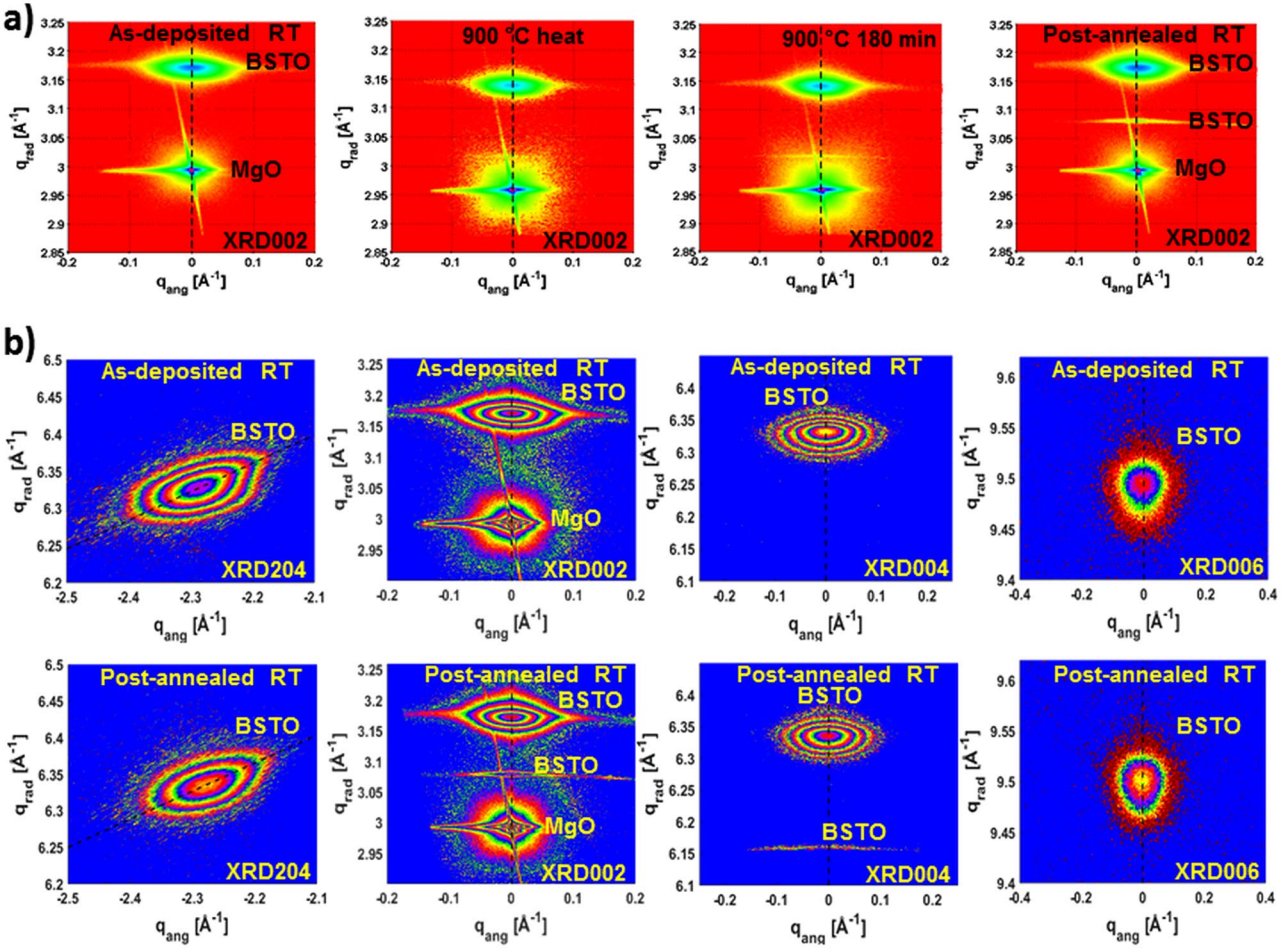
(**a**) 2D-RSMs of the XRD002 reflection corresponding to the as-deposited state, at *T* = *900* °*C* starting of the annealing, at *T* = *900* °*C* after *180 min* of annealing and in the post-annealed state. (**b**) 2D-RSM of the XRD204, XRD002, XRD004 and XRD006 reflections listed from left to right. The first row corresponds to the as-deposited state while the second row to the post-annealed state.

The 2D-RSMs of the symmetrical XRD002 and XRD004 reflections in the *as-deposited* state in Fig. (1a) show the presence of two main diffraction spots, corresponding to the MgO substrate and to the BSTO film.

Symmetrical x-ray diffraction (SXRD) and grazing incidence diffraction (GID) geometries are selectively sensitive to the vertical lattice strain and to in-plane strain components, respectively. Therefore, complementary XRD**002** and GID**200** reflections were sequentially recorded *in situ* during thermal treatment in order to separately characterize the out-of-plane and in-plane lattice components of the BSTO phases. Oppositely to XRD002 where diffraction spot is given by crystalline planes which are parallel to film surface, GID diffraction pattern is rather generated by the diffraction planes which are perpendicular to the film surface going across the complete film thickness. This makes GID a well suited method for an accurate determination of the in-plane lattice parameters despite of the limited penetration depth of *200 nm*. The grazing incidence x-ray beam increases the sensitivity and the reliability of the method to measure accurately the in-planes lattice parameters for the grown film independently of the penetrated depth.

**Figure 2:**
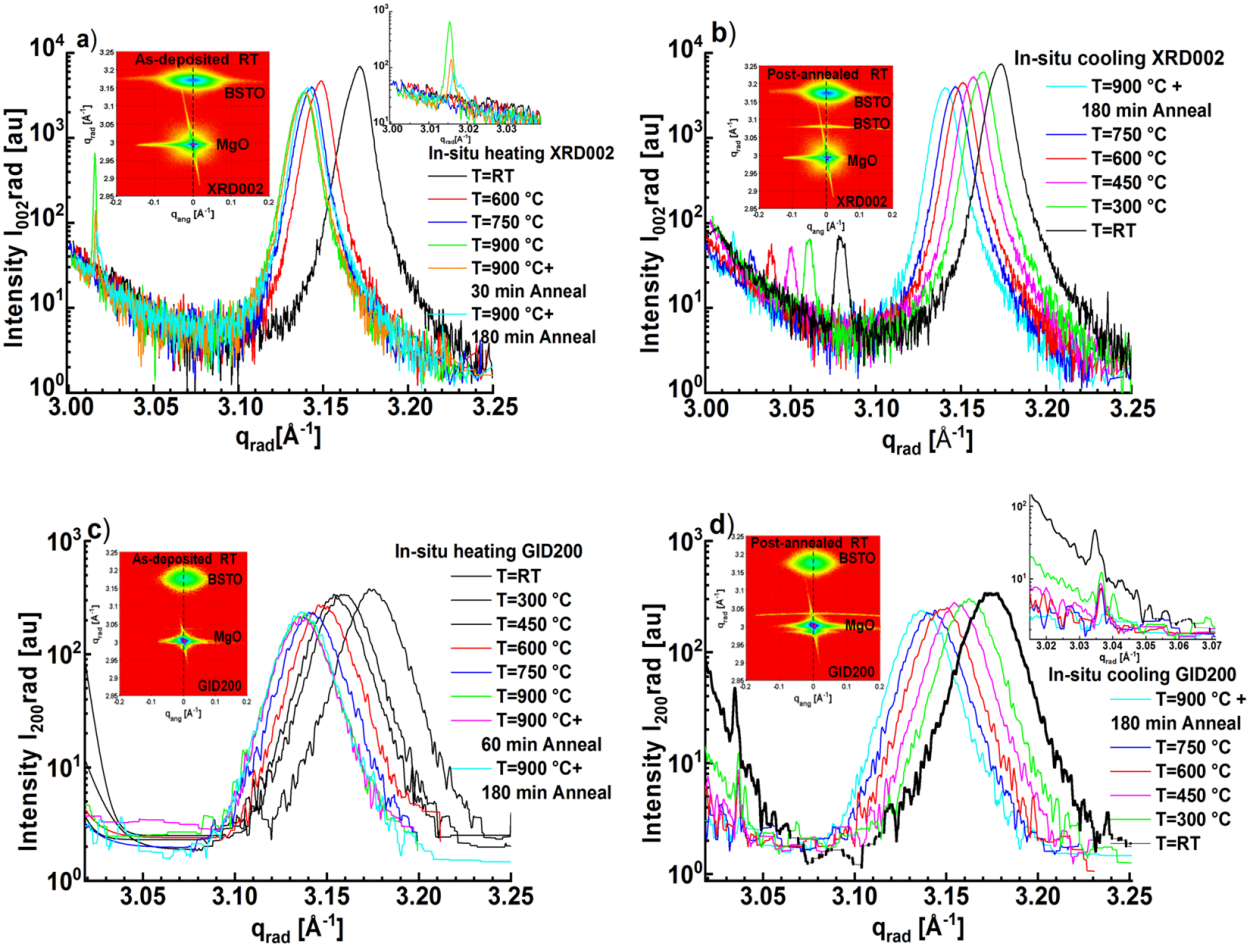
Diffraction radial profiles derived from the vertical scans at *q*_*ang*_ = *0* of the corresponding 2D-RSMs of XRD002 symmetric reflection acquired during the heating and the annealing steps (**a**) and during the cooling step (**b**). Diffraction radial profiles derived from the vertical scans at *q*_*ang*_ = *0* of the corresponding 2D-RSMs of GID200 grazing incidence diffraction acquired during the heating and the annealing steps (**c**) and during the cooling step (**d**).

From the measured 2D-RSMs of XRD002 and GID200 during the heating/annealing and the cooling phases, the radial diffraction profiles (parallel to the reciprocal lattice vector) were derived and illustrated in Fig. (2a–d), respectively.

In Fig. (2a,c), due to the thermal expansion, the BSTO peak position moves toward lower scattering vectors *q*_*rad*_ reflecting an expansion in the lattice parameters *c* and *a*. The intensity decrease of the BSTO peak during the heating phase can be attributed to the intensities losses by the thermal vibration. Oppositely, Fig. (2b,d) reveal a shift in the position of the BSTO peaks toward higher scattering vectors *q*_*rad*_, caused by a contraction in both lattice parameter components *c* and *a*, due to the negative thermal expansion during the cooling phase. Furthermore, it has been noted that the intensity of the BSTO peaks increases accordingly, due to the decrease of the thermal vibration.

As it can be shown in Fig. (1b) RSMs of main BSTO XRD spots are characterised by an asymmetry in their shapes along the radial direction, which can be interrelated with the presence of two BSTO phases, BSTO1 and BSTO2, having two slightly different out-of-plane lattice parameters *c1* and *c2*. In the following, we will label the according diffraction spots by BSTO (1, 2).

Contrary to the asymmetric shape of XRD002 reflections, all the corresponding BSTO (1, 2) radial diffraction profiles of the GID**200** reflections (see Supplementary Fig. (4)) are found to be symmetric. This indicates that the two BSTO (1, 2) phases of the film have different out-of-plane lattice parameters, but identical in-plane lattice parameters.

**Figure 3:**
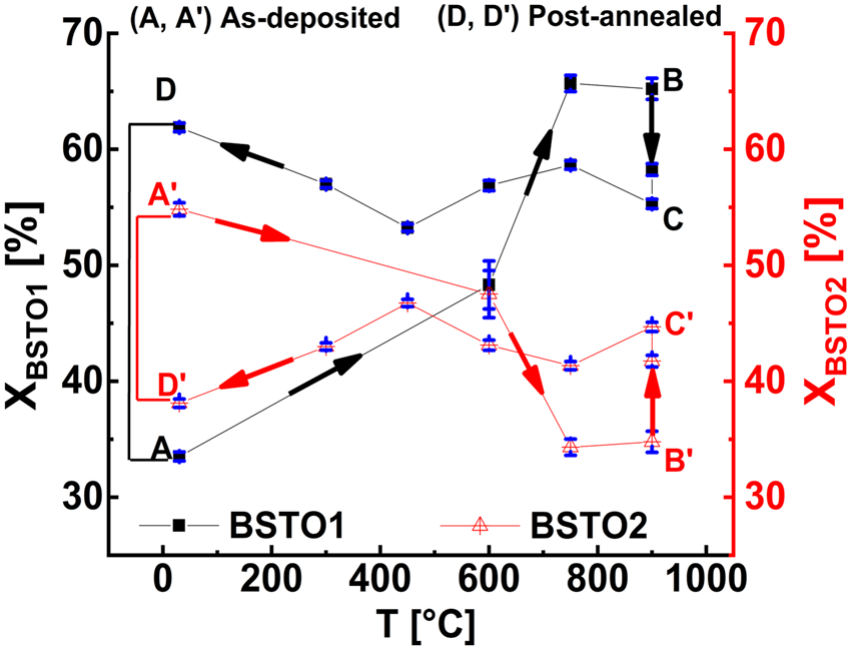
Variation of the BSTO1 and BSTO2 proportions *X*_*BSTO1*_ (solid black square) and *X*_*BSTO2*_ (open red triangle) with the temperature. [A-B] and [A’-B’] correspond to the heating step, [B-C] and [B’-C’] to the annealing step and [C-D] and [C’-D’] to the cooling step for the BSTO1 and BSTO2 respectively. A and D correspond to the state of the BSTO1 phase in the as-deposited and in the post-annealed states respectively while A’ and D’ correspond to the BSTO2 phase in the as-deposited and in the post-annealed respectively.

Moreover, we find the GID curve profiles shown in the Supplementary Figure (4) to stay very stable during thermal treatment where the asymmetric shape of the XRD002 reflections develops with thermal treatment. From the time evolution of the asymmetry in the BSTO (1, 2) peaks, we can conclude on the processes of post-growth phase transition, occurring during the different phases of thermal treatment. Indeed, a careful curve analysis of the BSTO (1, 2) peak shapes provide valuable insight into the evolution of the ratio of both phases as well as their vertical and lateral lattice components. All BSTO (1, 2) diffraction peaks of the XRD**002** and GID**200** reflections were accurately analysed using a fitting procedure based on a multi-start approach (see Supplementary Figures (3a–c) respectively). From the multi-peak analysis, the peak positions ***Q****c1* and ***Q****c2* as well as the other fitting parameters were derived for the two coexisting BSTO1 and BSTO2 phases and summarised in the Supplementary Tables (1a–c) for the heating, annealing and cooling phases. As consequence, the conversion behaviour between the BSTO1 and BSTO2 phases meaning the variation of the concentration of each phase, *X*_*BSTO1*_ [%] and *X*_*BSTO2*_ [%] was determined and plotted as function of the temperature in Fig. (3).

During the heating phase [A→B, A’→B’ in Fig. (3)] the proportion of the BSTO1 phase increases from about *33*.*52%* up to *65*.*68%*, accordingly, the concentration of the BSTO2 phase *X*_*BSTO2*_ [%] decreases from *54*.*82%* to *34*.*32%* as the temperature increases from room temperature up to *750* *°C* which is still below the growth temperature *Tg* = *850* *°C* (see Supplementary Tables (1a)) and Fig. (3).

It should be point out that the fitting of diffraction of profile XRD002 at room temperature RT shown in the Supplementary Figure (2a) demonstrates the existence of a residual phase which can be an impurity in the grown film with a percentage of *14*.*89%*. Th*e* concentration of the residual phase vanishes completely as the temperature becomes *750* *°C* (see column (10) in the Supplementary Tables (1a)).

Further increase of the temperature from *T*  = *750* *°C* (i.e. below *Tg* = *850* *°C*) up to *900* *°C* (i.e. above the *Tg* = *850* *°C*) does not alter significantly the ratio of the concentrations of BSTO1 and BSTO2 phases.

The annealing step [B→C] resp. [B’→C’] at *900* *°C* has contributed to a slight *10%* increase of the concentration of BSTO2 in expense of the BSTO1 phase.

During the cooling step [C→D] resp. [C’→D’], by crossing temperatures at the post-annealing lower than the growth temperature *Tg*, a slight recovery of the BSTO1 phase has taken place and the concentration *X*_*BSTO1*_ [%] has reached *61*.*89%* while the concentration *X*_*BSTO2*_ *[%]* has decreases to *38*.*10%* at room temperature (see column (11, 12) in the Supplementary Tables (1c)).

The content of the BSTO1 and BSTO2 phases were compared in the as-deposited and post-annealed states in the supplementary Table (2a) (line (6) and (7) in Table (2a)). Altogether, after annealing the phase dominance has moved from the BSTO2 to the BSTO1 phase, the latter representing with *X*_*BSTO1*_ = *61*.*89%* the major crystalline phase in the post-annealed state.

A study of the chemical composition of the films in the as deposited and post-annealed states by means of x-ray photoelectron spectroscopy (XPS) provides the XPS spectra of Ba atoms in the Supplementary Figures (1a,b). From the spectra and the integrated areas of Ba1 atoms and Ba2 atoms therein we deduce that the BSTO2 phase containing the Ba1 atoms and corresponds to the perovskite phase while BSTO1 consisting of Ba2 atoms and represents a non-perovskite one in the grown film in the as-deposited state. This effect was analysed and discussed in details by Rodrigues *et al*.^19^.

The 2D-RSMs corresponding to the post-annealed state of Fig. (1) and the radial curves in the onset of Fig. (2a,d) illustrate the appearance of an additional peak during thermal annealing. This new diffraction peak (for the XRD002 reflection located at *q*_*rad*_ =  *3*.*078* *Å*^−*1*^, indicates the formation of probably a new crystalline phase “BSTO3” having an out-of-plane lattice parameter *c3*  = *2* * *(2π/q*_*rad*_) = *4*.*08* *Å*. An attempt to interpret the chemical composition of the BSTO3 phase Ba_x_Sr_(1−x)_TiO_3_ from the measured lattice parameter (*c3*, *a3*) and from the known lattice parameters of BaTiO_3_ and SrTiO_3_ indicate that BSTO3 is a Ba-rich phase. This result correlates well with the XPS measurement given in the Supplementary Figure (1b), which also indicates the existence of regions with BaTiO_3_-like behaviour at the surface layer^19^.

### Evolution of residual strain during heat treatment

For the data evaluation, the MgO peak at room temperature was taken as a reference. From the relative shift of the Bragg peaks of BSTO (1, 2) with respect to that reference peak we conclude on the lateral and vertical lattice misfit between BSTO and MgO, and finally on the vertical and lateral lattice parameters *c* and *a* of the BSTO (1, 2) phases. In order to determine quantitatively the corresponding strain components, we need to refer to an unrelaxed lattice state. Here we assume for the relaxed (unstrained, strain-free) lattice states of both BSTO phases to have cubic lattice symmetry as it is described in details in section ***Data analysis procedur****e* by the two equilibrium parameter *a01* and *a02*.

The lattices parameters of the substrate and of the BSTO film expand during the heating and decrease during the cooling. Beside the reversible temperature-driven lattice expansion/contraction of the BSTO (1, 2) lattices, there might be other non-reversible influences related to impurities, oxygen vacancies and to further plastic strain relaxation due to thermal stress caused by differences in the thermal expansion coefficients of substrate and film lattices.

**Figure 4:**
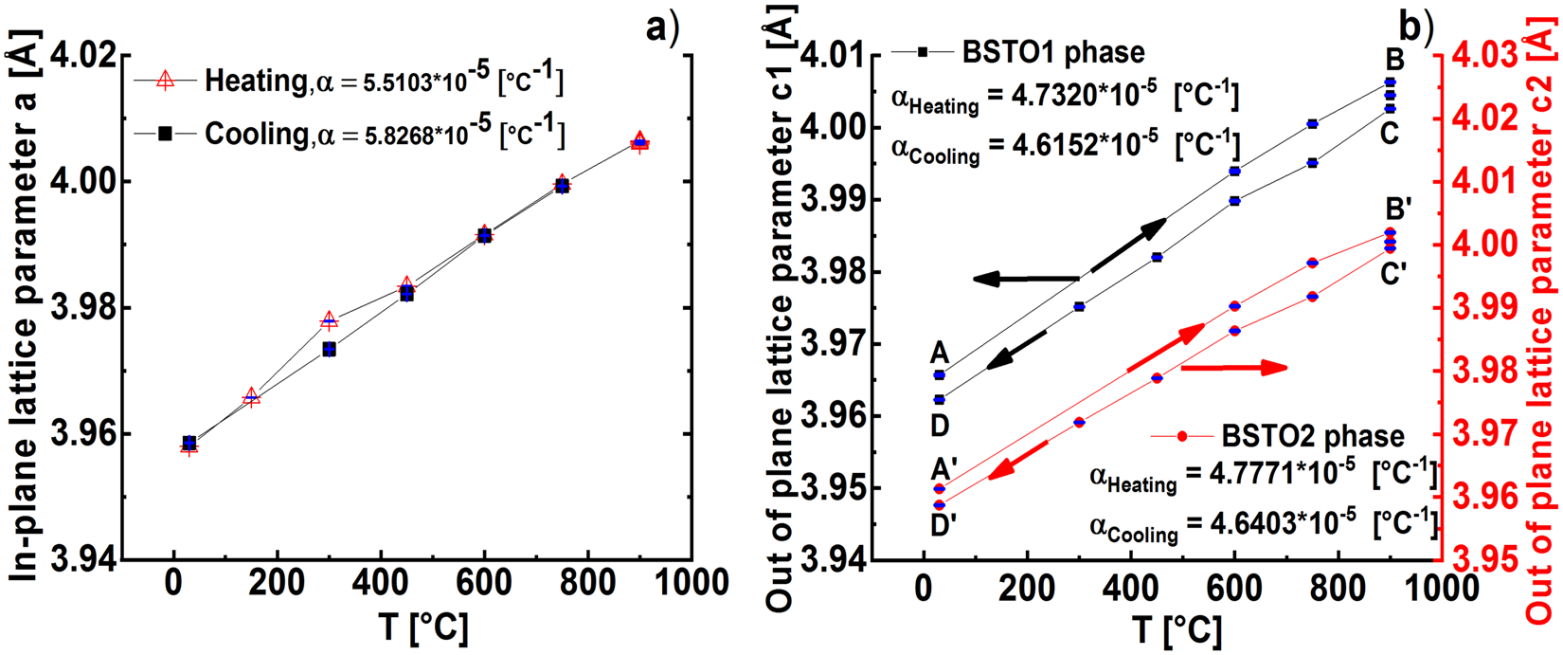
(**a**) The variation of the in-plane lattice parameter with temperature during the heating step (cross red triangle) and during the cooling step (solid black rectangle) of the BSTO1,2 phases. (**b**) Variation of the out-of-plane lattice parameters *c1* and *c2* corresponding the BSTO1 (solid black square) b and BSTO2 (solid red circle) phases respectively. [A-B] and [A’-B’] correspond to the heating step, [B-C] and [B’-C’] to the annealing step and [C-D] and [C’-D’] to the cooling step for the BSTO1 and BSTO2 respectively.

The variation of the in-plane lattice parameters with the temperature is quiet linear and nearly reversible (see Fig. (4a)). The observed effect will be certainly driven by temperature dependent lattice expansion/contraction. The variation of the out-of-plane lattice parameters *c1* and *c2* with the temperature is illustrated in Fig. (4b). The lattice parameters *c1* = 2*(2π/*Qc1*) and *c2* = 2*(2π/*Qc2*) of the BSTO1 and BSTO2 phases, respectively, were derived from the peak positions *Qc1* and *Qc2* of the BSTO1/BSTO2 phases and they were found to vary nearly linearly with the temperature. Again, the slopes of thermal lattice expansion during heating compared to cooling are nearly identical within each individual phase. Additionally, the comparison between the slopes of the two phases shows nearly no difference (see Fig. (4b)).

The out-of-plane lattice parameters *c1* and *c2* of the post-annealed state (D, D′) are lower than the ones of the as-deposited (A, A’). Since the post-annealing was performed with high oxygen pressure of *450 mTorr*, the oxygen vacancies will be filled and therefore the density of the vacancies will be reduced which leads to the contraction of the out-of-plane lattice parameter. Furthermore, there is a decrease in the peak intensity of the atoms O3 (i.e. binding energy of about *531.6 eV*) if we compare the spectra of the O 1 s recorded on the surface of the same BSTO film in the as-deposited state (c) and in the post-annealed state (d) in the Supplementary Figure (1). This enables us to conclude that the decrease in the lattice parameter is related with the decrease in the oxygen vacancies during the post-annealing in rich-O_2_ background. This not the case of the in-plane lattice parameter which is not affected by the oxygen vacancies but by the lattice mismatch with the MgO substrate

During the annealing phase, we observe a contraction of the vertical lattice components (i.e. out-of-plane lattice parameter), for BSTO1 by about *Δc1* = *0*.*005* *Å*, for BSTO2, by *Δc2* = *0*.*002* *Å*. However, there was no remarkable change recorded for the in-plane lattice components of the film, probably due to the influence of the MgO interface.

The behaviour of the lattice components *a* and *c* will influence the evolution of the residual strain components of the BSTO1 and BSTO2 phases with the temperature, determined with the assumption that the relaxed state corresponds to a cubic crystalline structure (see section ***Data analysis procedur****e*).

**Figure 5:**
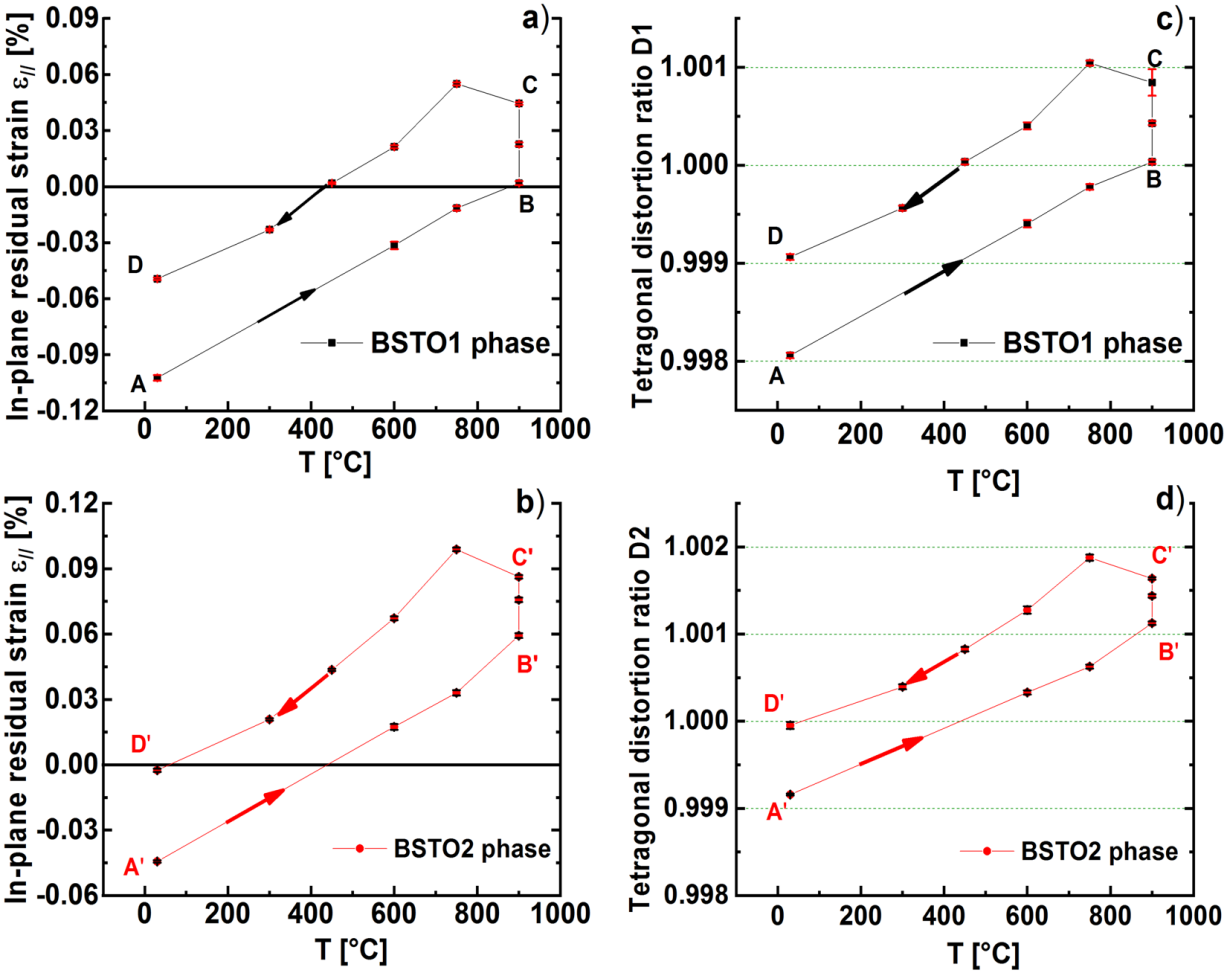
The variation of the in-plane residual strain [%] with temperature determined during the heating, annealing and cooling steps of the thermal treatment for (**a**) the BSTO1 phase and (**b**) the BSTO2 phase. The variation of the tetragonal distortion ratio with temperature determined during the heating, annealing and cooling steps of the thermal treatment for (**c**) the BSTO1 phase and (**d**) the BSTO2 phase. [A-B] and [A’-B’] correspond to the heating step, [B-C] and [B’-C’] to the annealing step and [C-D] and [C’-D’] to the cooling step for the BSTO1 and BSTO2 respectively.

Figure (5a,b) give the variation of the in-plane strain for the BSTO1 and BSTO2 phases. In Fig. (5c,d) we plot the corresponding ratios between the in-plane and out-of-plane lattice parameters and the tetragonal distortion ration *D* = *(a/c)* as a function of the temperature.

It should be emphasized that the absolute value of the determined strain are reliable as long as the assumption of the relaxed cubic state is valid for the MgO substrates and for the BSTO1/BSTO2 phases in the considered temperature range. However, the variation of in-plane strain and tetragonal distortion ratio illustrated in the figures as well as the differences between the BSTO1 and BSTO2-phases are unaffected by the above assumptions.

In all the figures, the segments [A-B] correspond to the heating step, [B-C] to the annealing step and [C-D] to the cooling step of the BSTO1 phase, respectively, [A’-B’], [B’-C’] and [C’-D’] to the BSTO2 phase. The values given by A and D (respectively A’ and D’) represent the status of the in-plane and out-of-plane strain for the BSTO1 phase (respectively for BSTO2 phase) in the as-deposited and in the post-annealed states.

#### Change of the residual strain in the annealing step [B-C] and [B’-C’] for the BSTO1 and BSTO2 phases:

 During the annealing step at *T *= *900* *°C* ([B-C] respectively [B’-C’]), for the BSTO1 and BSTO2 phases, the residual in-plane strain was tensile (ε_//_ > 0) and it has increased from *ε*_*//*_  = *0.0018* to *ε*_*//*_  = *0.0445* for the BSTO1 phase and from *ε*_*//*_ = *0.059* to ε_//_ = *0.0863* for the BSTO2 phase (see Fig. (5a,b)).

Even though, the in-plane lattice component *a* remains constant during annealing, a contraction of the out-of-plane lattice component *c* (see in Fig. (4b) the shifts from B to C for BSTO1 and B’ to C’ for BSTO2) has been measured. This has consequently led to a shift towards the tensile in-plane strain which could be illustrated by the curves given in Fig. (5a,b).

Annealing in flowing oxygen will have filled vacancies and hence has reduced the oxygen vacancy density which might explain the contraction in the lattice parameters for the BSTO1 phase by *Δc1* = −*0*.*004* *Å*, for the BSTO2 phase by about *Δc2* = −*0*.*0025* *Å*. A possible correlation between the decrease of the out-of-plane lattice parameter and the reduction of oxygen vacancies was already stated by Chang *et al*.^15^.

#### Change of the residual strain in the heating and in the cooling steps [A-B] and [B-D] for the BSTO1 respectively [A’-B’] and [B’-D’] for BSTO2:

The behaviour of the BSTO2 phase is slightly different from that of the BSTO1 phase. In fact, for BSTO2 during heating [A’-B’], a transition from compressive strain (*ε*_*//*_ < 0) to tensile strain (*ε*_*//*_ > 0) is detected at *T* = *450* *°C* (see Fig. (5b)), while the in-plane strain for the BSTO1 phase remains yet compressive (*ε*_*//*_ < 0) but increases also linearly with temperature (see Fig. (5a)). A similar transition phenomena was previously measured by Zhu *et al*.^5^ for the variation of the in-plane strain with oxygen pressure in Ba_0.5_Sr_0.5_TiO_3_ thin films epitaxially grown on [001] MgO. There, it has been explained as a result of the non-isotropic stresses caused by lattice mismatch which leads to the change of preferential oxygen vacancy sites between (0, *1/2*, *1/2*) and (*1/2*, *1/2*, 0) due to different activation energies.

The comparison of the in-plane strain in the as-deposited and in the post-annealed states for the BSTO1 and BSTO2 phases is given in the Supplementary Table (2) and illustrated in Fig. (5a,b) with the values *ε*_//_ (A) and *ε*_*//*_ (D) respectively *ε*_*//*_ (A’) and *ε*_*//*_ *(*D’*)*.

The post-annealing has induced a change of the compressive in-plane strain for the BSTO1 phase towards the tensile one from ε_//_ (A) varies from −*0*.*10%* to *ε*_*//*_ (D) = −*0*.*05%*. A similar effect has been measured for the BSTO2 phase as the in-plane strain varies from *ε*_*//*_ (A′*)* = −*0*.*04* to *ε*_*//*_ (D′*)* = −*0*.*0025*. In addition, by annealing, the tetragonal distortion of the BSTO1 crystalline phase decreases as the ratio *D*1 approaches *1* and varies from *D1* = *0*.*99806* to *D1* = *0*.*99906*.

The theoretical analysis of the strain-dielectric constant dependency for Ba_0.5_Sr_0.5_TiO_3_ at room temperature as performed by Chang *et al*.^15^ demonstrates that the increase in the compressive strain towards the tensile contributes in the improvement of the dielectric constant. Furthermore, they found out that the dielectric constant increases rapidly with tension (*ε*_*//*_ > 0) in a ferroelectric phase and decreases gradually with compression (*ε*_*//*_ < 0) in a paraelectric phase. This indicates us that the post-annealing step induces probably an enhancement in the dielectric constant as it is predicted theoretically and confirmed experimentally by Chang *et al*.^1,15^. Furthermore, our results show that the post-annealing causes a decrease in the deviation of tetragonal distortion of the BSTO1 and BSTO2 phases from the cubic symmetric as the ratios between *D1 and D2 get closer* to 1 (A → D see Fig. (5c) and *A*’ → *D*’ see Fig. (5d)).

## Conclusions and Discussions

In summary, our *in situ* XRD study demonstrate the occurrence of reversible processes such as reversible temperature-driven lattice expansion/contraction of the BSTO (1, 2) lattices and non-reversible phenomena such as temperature-induced phase transformation from BSTO1 to BSTO2 under possible influence of oxygen vacancies and plastic strain relaxation during the heating, annealing and cooling of the post-growth treatment. 2D-RSMs recorded with high resolution diffraction in XRD and GID geometries were proven to be sensitive and a useful method to follow up the changes in the shape of the radial diffraction profiles. These have allowed us to determine the abundance of the BSTO1 phase in accordance with the BSTO2 phase and to monitor the temperature induced modifications occurring in the heating and in the cooling steps.

We observe the nearly identical slopes for lattice parameter evolution during heating and cooling, but slightly different slopes for both BSTO-phases. For both BSTO-phases, during the annealing phase, an increase of the vertical lattice misfit with respect to MgO is observed, where the lateral misfit remains constant.

We observed a transfer of phase dominance from BSTO2 in the as-deposited to BSTO1 phase in the post-annealed state and a change from a compressive strain towards tensile strain during annealing.

The cooling step from the annealing temperature to room temperature RT causes changes in the out-of-plane and in the in-plane lattice parameters of both phases. This would consequently affect the residual strain in the film as well as the tetragonal distortion of the crystal. The strain due to the thermal expansion difference between the film and the substrate affects the structure of the as-deposited film during cooling after the annealing processing step to RT. The resulting strain in the film at room temperature affects the microwave dielectric properties of the films. Our study has revealed a transition in the in-plane residual strain from compressive strain at room temperature to tensile strain at the annealing temperature similar to the transition record by Zhu *et al*.^5^ by varying the oxygen pressure. This interesting phenomenon emphasize that the annealing process could be used as method for the strain-engineering in BSTO grown on MgO which explains the improvement recorded in the ferroelectrics properties mentioned in former studies. Besides the changes in the residual strain induced during the cooling phase, a post-growth was demonstrated by the increase in the diffracted intensity.

## Methods

### PLD growth of Ba_0.5_Sr_0.5_TiO_3_ on MgO

Ba_0.5_Sr_0.5_TiO_3_ BSTO thin films were grown on MgO (100) substrates by pulsed laser deposition using a frequency quadrupled (266 *nm*) Nd:YAG laser operated at a repetition rate of *10 Hz*. Pulse duration of *5* *ns* and a single pulse energy of *63 mJ*/pulse at the target was used. The laser was focused to a spot with a size of *2* × *1* mm^2^ which leads to a fluence of *3*.*15* *J/cm*^2^. The average laser power is *0*.*63* *W* at *10* *Hz*.

The growth conditions and the post-thermal treatment have been performed as follows: As a first step, the BSTO film of *760* nm thickness was grown on the MgO substrate using a (BST, x = *0.5*) target. The thickness of the film was determined from the transmission electron microscopy TEM, not shown here. The target was rotated with 8 rpm during the ablation process. The substrate was positioned 4 *cm* away from the target and heated to the growth temperature *Tg* = *850* *°C* using a laser heater with an integrated pyrometer for temperature sensing by an infrared diode laser with a spot size diameter of approximately 10 *mm*. In order to determine accurately the temperature of the film, the sample holder was additionally equipped with a thermocouple. The growth was carried out with an oxygen background with a pressure of *0*.*0999 mbar* (i.e. *9*.*99* *Pa*, *75 mTorr*). The sample was cooled to room temperature with the cooling rate of *10* *°C/min*. The growth process was previously studied by *in situ* XRD measurements, performed on other BSTO films grown but with comparable growth condition^17^.

As a second step, a post-thermal treatment was applied to the BSTO film in oxygen background with a pressure of *450 mTorr*, where the sample is heated with a heating rate of *150* *°C/min* from room temperature to the annealing temperature of *T* = *900* *°C* which afterwards was set constant for a duration of *180* *min*. Finally, the film was cooled with a cooling rate of *150* *°C/min*.

In the present study, *in situ* x-ray diffraction experiments were carried out during the post-growth thermal treatment.

### Description of the set-up for *in situ* synchrotron high resolution x-ray diffraction at the NANO beamline

The synchrotron x-ray diffraction measurements were carried out at the NANO beamline of the Institute of Photon Science and Synchrotron Radiation at KIT (Karlsruhe, Germany). The beamline is dedicated for high resolution X-ray diffraction, surface and interface scattering. In the experiment, the x-ray beam was focused to *200* *µm* horizontally and *80* *µm* vertically at the sample position by using the two last cylindrical mirrors of the beamline optics. The estimated horizontal and vertical divergences are *0.315* × *0.2* *mrad*^*2*^ at full width at half maximum (FWHM). X-ray diffraction experiments were carried out at photon energy of 17 *keV* and with *10*^*−4*^ energy resolution realized by a Si (111) monochromator.

The *in situ* x-ray characterization during the thermal treatment was performed by using an *in situ* PLD growth and characterization chamber which could be used for growth as well as annealing at temperature up to *1050* *°C* for up to *24* hours (see^16^ for a detailed description of the functionalities). The chamber is equipped with an entrance and an exit Beryllium window to permit *in situ* x-ray diffraction studies. The incidence angle is limited to *55°*, as defined by the opening angle of the entrance window. It has the unique feature to be portable from the laboratory to the beamline and to be coupled with the heavy duty diffractometer of NANO enabling time resolved measurements of coplanar and non-coplanar high resolution diffraction during growth, heating, annealing and cooling steps.

### Data acquisition procedure

**Figure 6:**
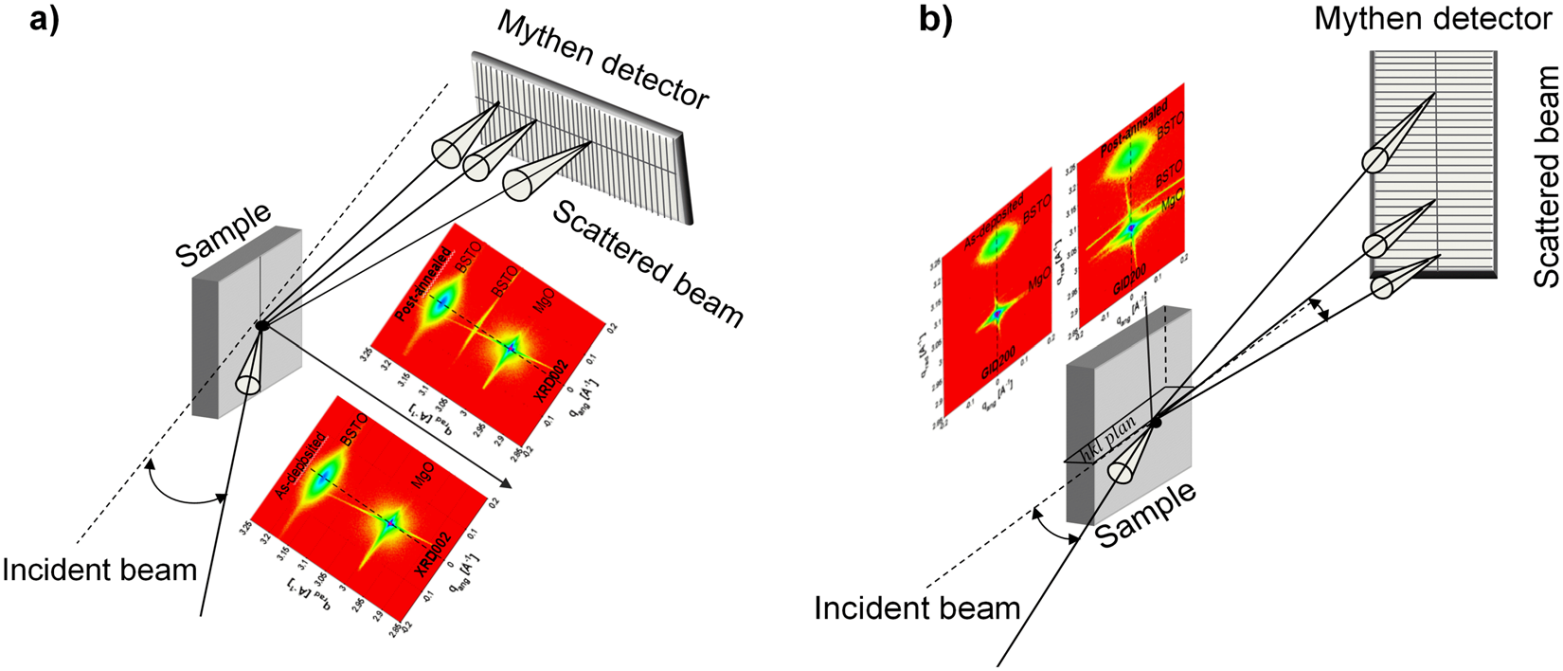
(**a**) Schematic presentation of the diffraction geometry for the acquisition of the 2D-RSMs of the symmetric XRD002 reflection and asymmetric XRD204 reflection. The sample is placed vertically rotated with respect to the incident beam. A microstrip solid-state detector Mythen 1 K, having *1280* channels which are spread within the horizontal diffraction plane. (**b**) Schematic presentation of the grazing incidence diffraction geometry for the acquisition of the 2D-RSMs of GID200 reflection. The sample is rotated around the surface normal to bring the (hkl) plane in diffraction. A microstrip solid-state detector Mythen *1 K*, having *1280* channels which are spread within the vertical diffraction plane.

Figure (6a,b) illustrate the diffraction geometries used for the acquisition of the (002) symmetric reflection XRD002 and of the (200) grazing incidence diffraction GID200 respectively. For both cases, the sample surface was mounted on a hexapod vertically with respect to the incident beam inside the PLD chamber.

All x-ray data were recorded with a microstrip solid-state detector Mythen 1 K, featuring *1280* channels with a channel size of *50* *µm* and a point-spread function of one channel. The channels are spread within the horizontal diffraction plane for measuring the XRD002 reflection (see Fig. (6a)) and within the vertical diffraction plane for the acquisition of the GID200 reflection (see Fig. (6b)).

The 2θ Bragg angle, defined as the angle between the direct and the diffracted beams, can reach up to *110°* in this setup thanks to the size of Beryllium exit windows. The *0*.*002°* angular resolution of the diffracted beam was predefined by the channel size of *50* *µm* and the distance between sample and the detector equal to *1406*.*9* *mm*.

2D-RSMs of the XRD002 reflection were recorded by means of rocking curve scans which were carried out by rotating the whole chamber around the vertical axis in Bragg conditions, while the detector was kept at the respective Bragg diffraction angle in the horizontal diffraction plane (see Fig. (6a)). The sample surface was aligned with respect to the direct incident beam using the six degrees of freedom of the hexapod.

2D-RSMs of GID200 reflection were acquired by rotating the sample mounted on the hexapod around its normal while keeping the detector at the respective Bragg diffraction angle in the vertical diffraction plane (see Fig. (6b)). The grazing incidence angle of the incident beam with respect to the diffracting surface was given by the rotation of the PLD chamber with an angle of 0.2 degree around the vertical axis of the diffractometer.

The 2D-RSMs were recorded in the heating step for the temperatures *T* = *RT*, *600* *°C*, *750* *°C* and *900* *°C* (see Fig. (2a)) while every *150* *°C* during the cooling step (see Fig. (2b)).

The 2D-RSMs of GID200 reflection were recorded in steps of *150* *°C* during the heating and the cooling phases.

During the annealing step at *900* *°C*, the 2D-RSMs were measured after an annealing time of *30* *min*, *60* *min* and *180* *min*.

The 2D-RSMs of the XRD002 and GID200 reflections were recorded sequentially and the acquisition time for each 2D-RSM was about *2* *min*. The 2D-RSMs were calculated from angular coordinates to reciprocal space coordinates using self-developed program based on Matrix transformation in Matlab language^18^. A correction of the intensity with the decay of the electron current during the measurement as well as the background subtraction was taken into consideration to get an accurate variation of the scattered intensity during the thermal treatment.

Figure (6a,b) show also two 2D-RSMs of the XRD002 reflection and two 2D-RSMS of the GID200 reflection respectively. The latter correspond to the as-deposited and to the post-annealed states.

In order to clearly demonstrate the characteristic asymmetry in the XRD spots due to the BSTO (1, 2) phases, higher order reflections XRD004 and XRD006 were recorded in the as-deposited and in the post-annealed states. In Fig. (1b), the first row illustrates the 2D-RSMs of the asymmetric reflection XRD204 and the higher order reflections XRD004 and XRD006 in the as-deposited state. The second row shows the 2D-RSMs which belong to the post-annealed state.

### Data analysis procedure

The acquisition of 2D-RSMs present several advantages in comparison to the standard rocking curve scan. It enables us to get different cuts corresponding to different directions in the reciprocal space, thus permitting us to reliably extract numerous structural features like crystalline sizes parallel and normal to the sample surface, residual strain in the grown film, crystal distortion and defects.

From each recorded 2D-RSM of the XRD002 reflection during the thermal treatment procedure, a vertical scan at *qang* = 0 (see Fig. (1)), named in this manuscript diffraction radial profile, was derived to determine the out-of-plane lattice parameters of the existing phases in the grown films. All the diffraction radial profiles determined from 2D-RSMs of the XRD002 reflection during the whole treatment process are displayed in Fig. (2a,b).

These diffraction radial profiles of the XRD002 reflection were fitted to a linear combination of Voigt profiles. A multi-start approach guaranties that the resulting best fitted profiles become independent from the starting parameters. The quality of the fit for all the diffraction profiles is measured by the reduction of the chi-square to a value in the order of 10^−*5*^. All the statistical errors of the determined structural parameters are included in the Supplementary Tables (1) and (2).

The choice of the number of diffraction peaks, i.e. the number of Voigt profiles, for the fitting procedure is related with the number of phases present in the grown film. X-ray photon spectroscopy XPS study^19^ was described in details in the Supplementary Note 1. In the Supplementary Figure (1a), the XPS spectra of Ba atoms at the surface layer of as-deposited film, have revealed the existence of two phases in the as-deposited state of the film: perovskite phase (Ba1) and non-perovskite phase (Ba2).

All fitting results corresponding to the heating and to the cooling steps *Qcres*, *Ares*, *Qc1*, *FWHM1*_*rad*_, *A1*, *Qc2*, *FWHM2*_*rad*_, *A2*, *X*_*BSTO1*_ and *X*_*BSTO2*_ with their statistical errors are summarised in the Supplementary Tables (1a), (1b) and (1c) where the *Qcres*, *Qc1*, *Qc2* are the position of peak BSTOres, peak BSTO1 and peak BSTO2 respectively (see columns 2, 4 and 7 in Supplementary Tables (1a–c)).

BSTOres corresponds to the residual phase of BSTO since it represents the lowest proportion calculated from the fitting parameters. BSTO1 correspond to the crystalline phase having the peak position at lower and BSTO2 at higher scattering vector *Q* values. It is not possible from the diffraction profile to determine the corresponding chemical phases in similar manner as in the XPS study.

*FWHM1*_*rad*_, and *FWHM2*_*rad*_ are the FWHM of the diffraction radial profiles of BSTO1 and BSTO2 peak profiles (given in column 5 and column 8 in Supplementary Tables (1a–c)).

*X*_*BSTOresr*_ *X*_*BSTO1*_ and *X*_*BSTO2*_ (column 10, 11 and 12 in Supplementary Tables (1a), (1b) and (1c)) are the proportion of BSTOres, BSTO1 and BSTO2 phases respectively, determined from the integrated areas *Ares (column 3)*, *A1* (column 6) and *A2* (column 9) in the Supplementary Tables (1a), (1b) and (1c)) of the diffraction radial profiles BSTOres, BSTO1 and BSTO2 respectively as follow:

$${X}_{BSTOres}=Ares/(Ares+A1+A2),{X}_{BSTO1}=A1/(Ares+A1+A2)\,{\rm{and}}\,{X}_{BSTO2}=A2/(Ares+A1+A2)$$

From the peaks position, *Qc1* and *Qc2*, the corresponding out-of-plane lattice parameter *c1* and *c2* were determined by *c1* = 2*(*2π/Qc1*) and *c2* = 2*(*2π/Qc2*) (column 13 and 14 in the Supplementary Tables (1a), (1b) and (1c))).

In summary, the structural parameters derived from 2D-RSMs of the XRD002 reflections are inserted in columns 2 up to 14 in the Supplementary Tables (1a–c).

- Similar data processing was applied on the individual recorded 2D-RSM of the GID200 reflections where the penetration depth was limited to *200* nm due to the grazing incidence angle of *0*.*2 degrees*. A linear cut were applied: a vertical along the *q*_*rad*_. The radial profiles which are displayed in Fig. (2c,d) correspond to the heating and cooling steps, respectively. Oppositely to the XRD002 diffraction profiles, the GID200 profiles were rather symmetric and they could be accurately fitted with only one Voigt peak function. As a result, the in-plane lattice parameter *a* = 2*(2π/Qa) was calculated (column 16 in the Supplementary Tables (1a–c)) from the peak position *Qa* ((column 15 in tables (1a), (1b) and (1c)).
- In order to determine the residual strain, we used the equilibrium (strain-free) lattice parameter *a0*^20^ instead of the lattice parameter of the bulk Ba_0.5_Sr_0.5_TiO_3_ (i.e. *3.9475 Å*). In fact, the presence of oxygen vacancies in the film and the lattice mismatch between the deposited layer and the substrate make the equilibrium (strain-free) lattice parameter of BST thin film different from the ideal bulk one. In this case, the ideal bulk lattice parameter (*3.947 Å*) should not be used in the film strain calculation^15^. The equilibrium lattice parameter *a0* is calculated from the measured in-plane lattice parameter *a* from peak position of the GID200 reflection and from the out-of-plane lattice parameter as it is derived from the peak position of the XRD002 reflections as follow^21^:

Since the film contains BSTO1 and BSTO2 phases having different out-of-plane lattice parameters *c1* and *c2*, two equilibrium parameter *a01* and *a02* will be derived:

$$a01/02=\frac{[c1/2+\,2\ast (\frac{{c}_{12}}{{c}_{11}})\ast a\,]}{[1+2\ast (\frac{{c}_{12}}{{c}_{11}})]}$$

where *c*_*ij*_ are the elastic constants of BSTO *c*_11_ = *3*.11*645* × *10*^11^ *N/m*^2^ and *c*_12_ *= 1*.*39805* × *10*^11^ N*/m*^2^ which are obtained by averaging the elastic constants of bulk SrTiO3 and BaTiO3 single crystals.

The in-plane residual strain *ε*_//_ and the out-of-plane residual strain in the films *ε*_*⊥*_ are expressed for the BSTO1 phase as:

$${\varepsilon }_{//}=\frac{[a-a01]}{a01}\,and\,{\varepsilon }_{{\perp }}=\frac{[c1-\,a01]}{a01}$$

In similar way the in-plane residual strain *ε*_//_ and the out-of-plane residual strain in the films *ε*_⊥_ are expressed for the BSTO2 phase as:

$${\varepsilon }_{//}\,=\frac{[a-\,a02]}{a02}\,and\,{\varepsilon }_{{\perp }}=\frac{[c2-a02]}{a02}$$

The in-plane and out-of-plane residual strains were determined from the *in situ* measurement of the lattice parameters *a*, *c1* and *c2* during the heating, annealing and the cooling steps.

## Electronic supplementary material

Supplementary Information
