# Supplementary material for: Real time in situ x-ray diffraction study of the crystalline structure modification of Ba0.5Sr0.5TiO3 during the post-annealing
Source: Sci Rep. 2018 Aug 10;8:11969. doi: 10.1038/s41598-018-30392-y (PMC6086881; doi:10.1038/s41598-018-30392-y)
Supplement: Supplementary file 1 — Supplementary Information [file 41598_2018_30392_MOESM1_ESM.pdf]

Supplementary information

Real time *in situ* x-ray diffraction study of the crystalline structure modification of $Ba_{0.5}Sr_{0.5}TiO_3$ during the post-annealing

Sondes Bauer*[1], Adriana Rodrigues[1], Tilo Baumbach[1,2]

Institute for Photon Science IPS

[1] Institute for Photon Science and Synchrotron Radiation IPS, Karlsruhe Institute of Technology KIT, Hermann-von-Helmholtz-Platz 1, D-76344 Eggenstein-Leopoldshafen, Germany

[2] Laboratory for Application of Synchrotron Radiation LAS, Kaiserstraße 12 76131 Karlsruhe, Germany

*Contact person: sondes.bauer@kit.edu

Supplementary Note 1

X-ray Photoelectron Spectroscopy (XPS) measurements were performed in the ANKA UHV analysis Laboratory using a Phoibos 150 Analyzer and an unmonochromated XR-50 Mg Kα X-ray source from SPECS. The angle between the analyzer and the X-ray source was fixed to 45°. The base pressure inside of analysis chamber was about 3 x 10^{-10} mbar. All measurements were done at room temperature with pass energy set at 20 eV and an energy step at 0.05 eV. The deconvolution of the peaks was performed by the Fityk software using Voigt profiles and a linear background [1].The binding energy was calibrated using the Au $4f_{7/2}$ (84.00 eV) photoemission line of a corresponding reference material [2]. Due to the charging effect, the adventitious C 1s (284.5 eV) was required as an additional calibration of the binding energy [3]. The spectra of the Ba 3d recorded on the surface of BSTO film in the as-deposited state is shown in the supplementary figure (1a). Each of the Ba $3d_{3/2}$ and Ba $3d_{5/2}$ can be fitted by two peaks. The lower binding energy of the spin-orbit-split (SOS) pair at $3d_{3/2}$ BE_{Ba1} = 793.28 eV and $3d_{5/2}$ BE_{Ba1} = 777.95 eV, denoted as Ba1 is assigned to Ba1 atoms in perovskite lattice. The higher binding energy SOS pair at $3d_{3/2}$ BE_{Ba2} = 794.85 eV and $3d_{5/2}$ BE_{Ba2} = 779.52 eV, denoted Ba2, assigned to Ba2 in different chemical environment. Similar binding energy values were recorded for Ba1 and Ba2 by Baniecki *et al* [4]. The energy separation between Ba1 and Ba2 was 1.5 ± 0.1 eV.

Supplementary figures (1a, 1b) compare the XPS spectra of Ba 3d in the as-deposited and in the post-annealed states. Upon annealing, the binding energy of Ba atoms was shifted and the shape of the profiles was changed. A new phase has appeared leading to the detection of Ba peak, denoted as Ba3 with binding energy BE=781.75 eV due to regions with BaTiO3-like behavior as it is explained by Rodrigues *et al* [5] and Ba4 atoms, which probably corresponds to a Ba-O bond in the relaxed surface phase.

Supplementary figures (1c, 1d) compare the XPS spectra of the O 1s in the as-deposited and in the post-annealed states (1d). The lower binding energies (BE_{O1} = 528.65 eV, BE_{O1} = 528.88 eV) and (BE_{O2} = 530.55 eV, $BE_{O2=}$ 530.60 eV), labelled as O1 and O2 respectively, are assigned to O^{2-} ions in the BSTO phases. While the O3 atoms with (BE_{O3} = 531.60 eV, BE_{O3} = 531.85 eV) and O detected at (BE_{O} = 533 eV BE_{O} = 533.30 eV), are attributed to an oxidation state O^{x-} ($0<x<2$), corresponding to chemisorbed species as well as to oxygen vacancies and to the non-perovskite structure of BSTO. The O4 atoms with a binding energy BE_{O4} = 527 eV corresponds to the Sr-O bond in the SrO oxide component.

Supplementary Note 2

Supplementary Figure (2a) illustrates the 2D-RSMs of the symmetric XRD002 reflection over the three post-thermal treatment steps: heating, annealing and cooling. The first row starts with the 2D-RSM at room temperature and ends with 2D-RSM at the annealing temperature *900 °C*. The first three 2D-RSMs of the second row correspond to the annealing step with duration of *180 min*. The cooling step starts with the last 2D-RSM of the second row at *750 °C* until the next-to-last 2D-RSM at 300 °C.

Supplementary Note 3

During the heating phase (see supplementary figure (3a)), the diffraction profile of the XRD002 reflection is asymmetric and it could be fitted by three Voigt profiles (blue for BSTOres, magenta for BSTO1 peak and green for BSTO2 peak) indicating the presence of three different crystalline phases. As the temperature increases, the intensity of the BSTO1 peak increases on expenses of the intensity of BSTOres and BSTO2 peak, leading to the disappearance of the BSTOres phase at *T= 750 °C*.

The annealing phase (see supplementary figure (3b)) was characterised by a slight increase of the intensity BSTO2 peak on the expenses of the intensity of BSTO1 peak.

During the cooling step, the diffraction radial profiles of BSTO, shown in see supplementary figure (3c), were fitted by two peaks because of the profile asymmetry, originating from the existence of two crystalline phases.

Supplementary Note 4

All the corresponding BSTO (1,2) radial diffraction profiles of the GID200 reflections (see supplementary figure (4)) are found to be symmetric. This indicates that the two BSTO (1, 2) phases of the film have different out-of-plane lattice parameters, but identical in-plane lattice parameters. Moreover, we find the GID curve profiles shown in the supplementary figure (4) to stay very stable during thermal treatment where the asymmetric shape of the XRD002 reflections develops with thermal treatment.

Supplementary Note 5

Diffraction radial profiles corresponding to three different temperatures RT, T=600 °C and 750 °C recorded during heating (black) and during cooling (red line) of a) GID200 and b) XRD002.

No reversibility recorded for GID200 reflections while the XRD200 profiles corresponding during heating and cooling to a certain temperature are shifted for the same chosen heating and cooling rate of 150 °C/min. This would lead to a hysteresis in the variation of the out-of-plane lattice parameter with the temperature as it could be demonstrated in figure (5b).

Supplementary figures

Supplementary figure (1)

The XPS spectra of the Ba 3d and O1s recorded on the surface of BSTO film in the as-deposited state (a, c) and in the post-annealed state (b, d).

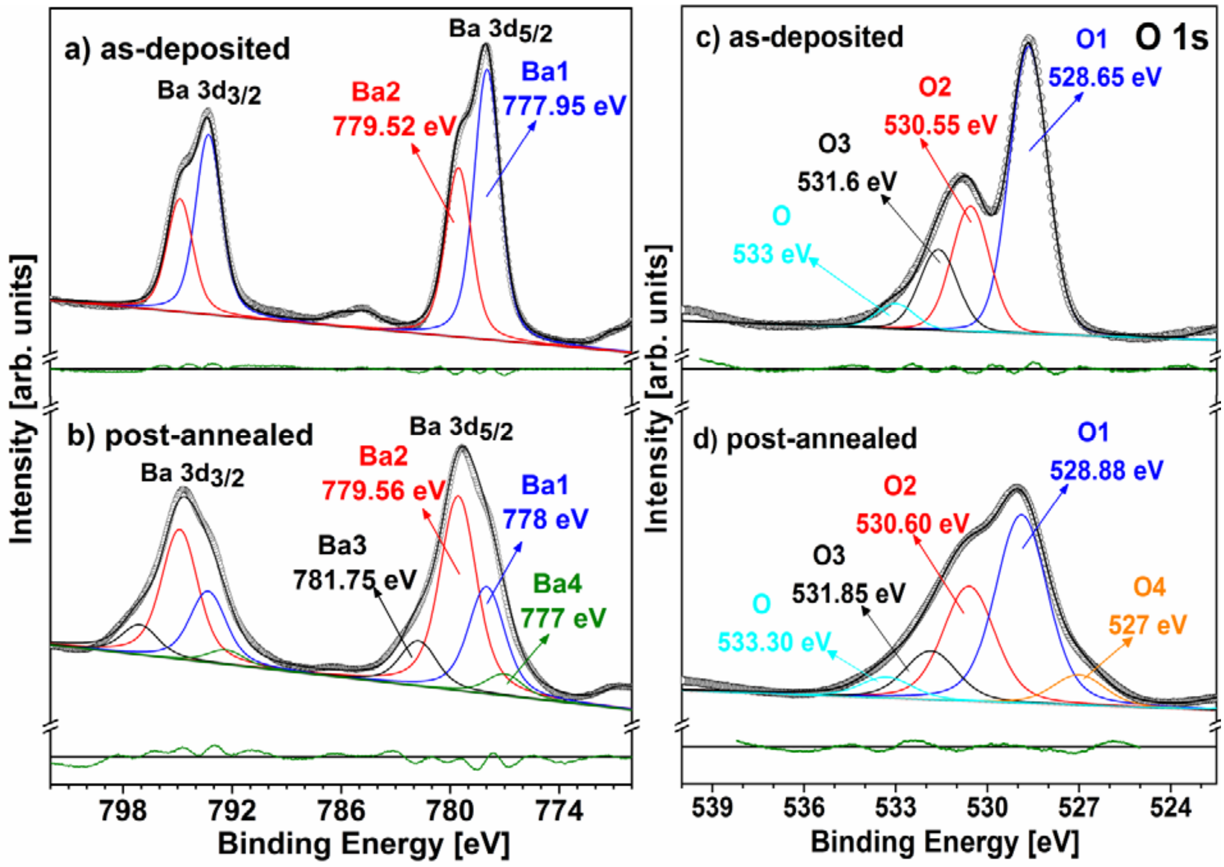

Supplementary figure (2)

2D-RSMs of the XRD002 reflection. The first row corresponds to the heating step. The first three 2D-RSMs of the second row correspond to the annealing step and the third row represents the 2D-RSMs recorded during the cooling step.

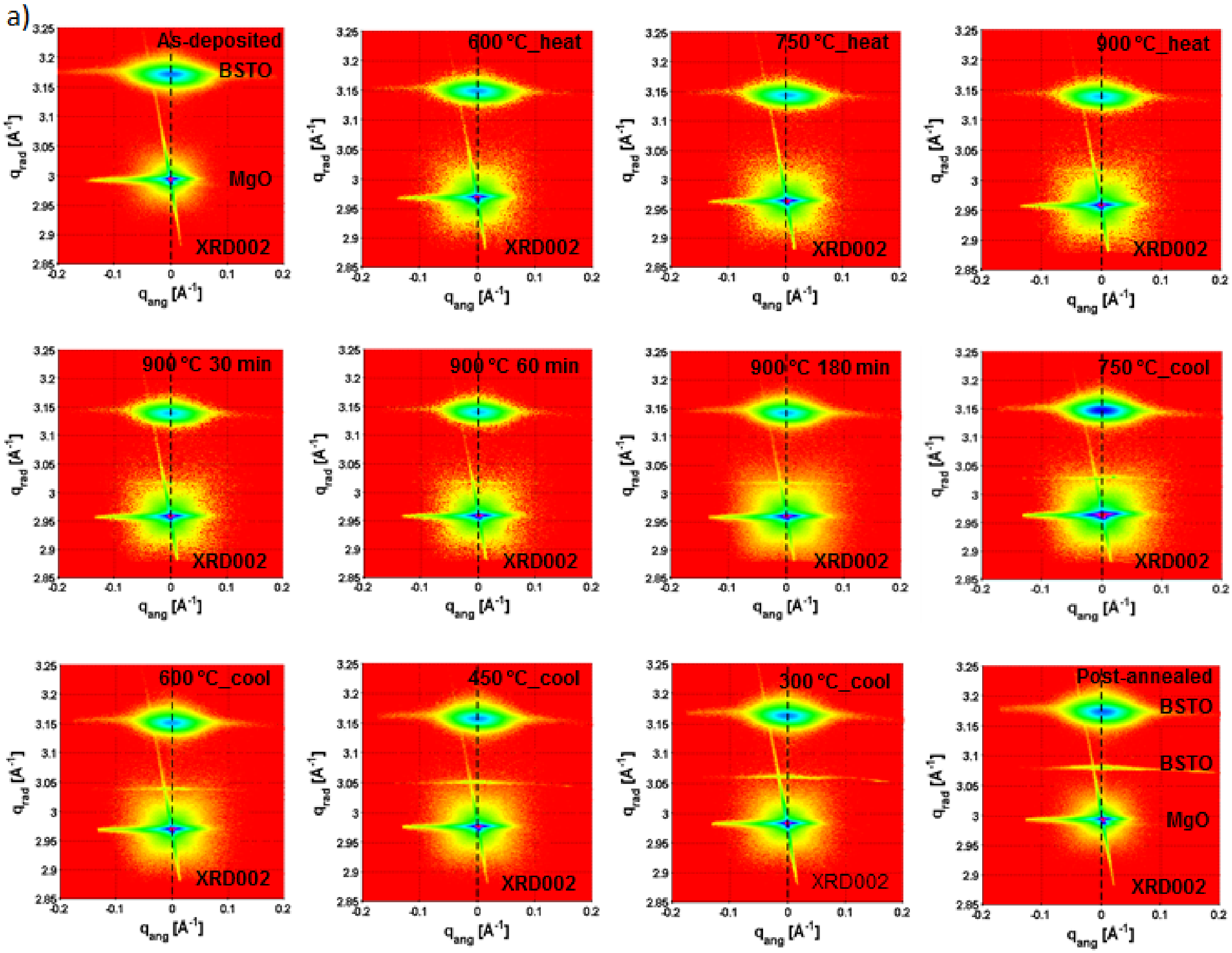

Supplementary figure (3)

Diffraction radial profiles of XRD200 symmetric reflection accompanied with the fitting curves using multi-start approach. The magenta-purple curves correspond to the BSTO1 phase while the green curves are of the BSTO2 phase, the blue for the BSTOres and the red is the fitting curves. The profiles were determined (3a) during the heating step, (3b) during the annealing step and (3c) during the cooling step.

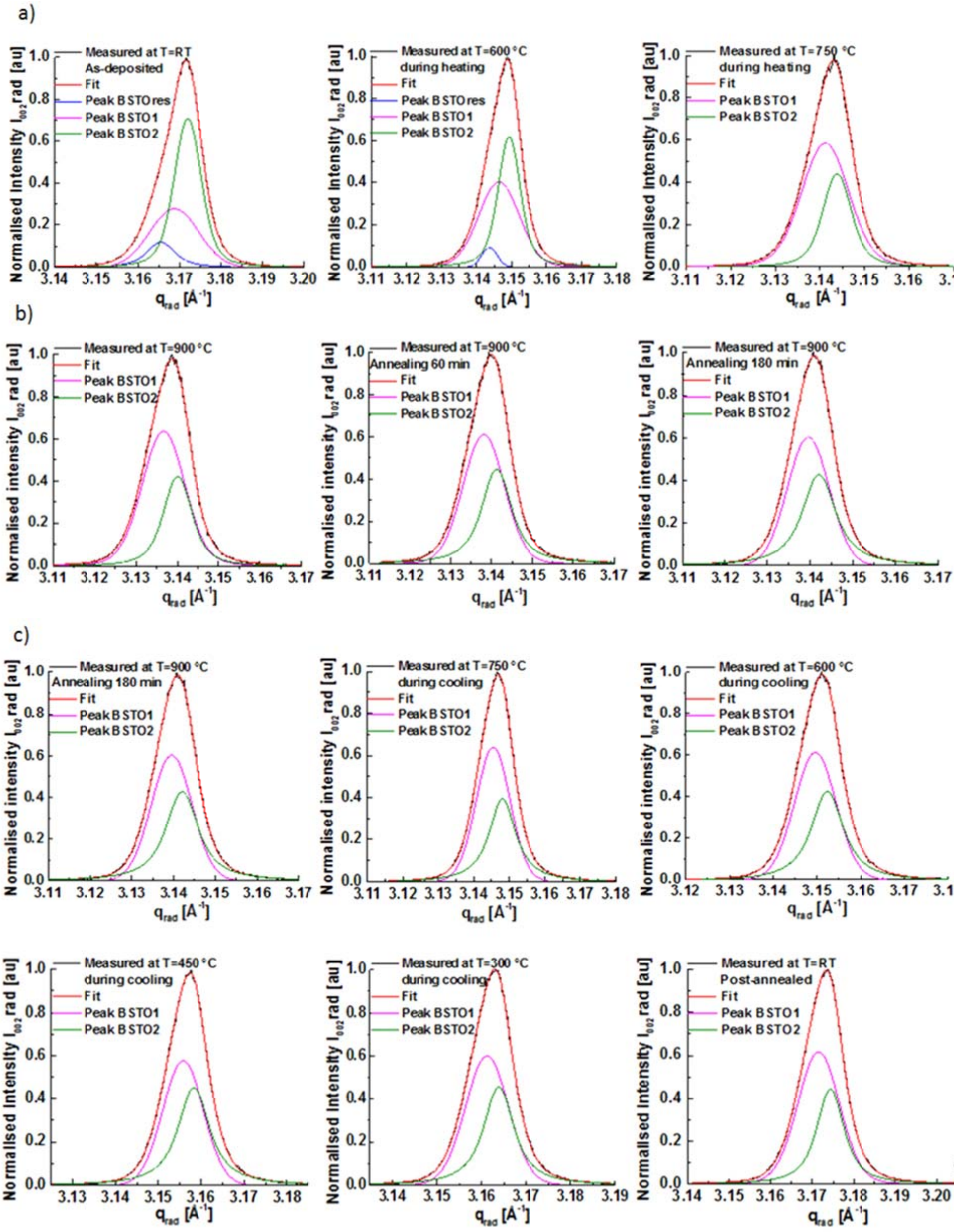

Supplementary figure (4)

Diffraction radial profiles of the GID200 reflection accompanied with the fitting curves using multi-start approach. The profiles were determined (4a) during the heating step, (4b) during the annealing step and (4c) during the cooling step.

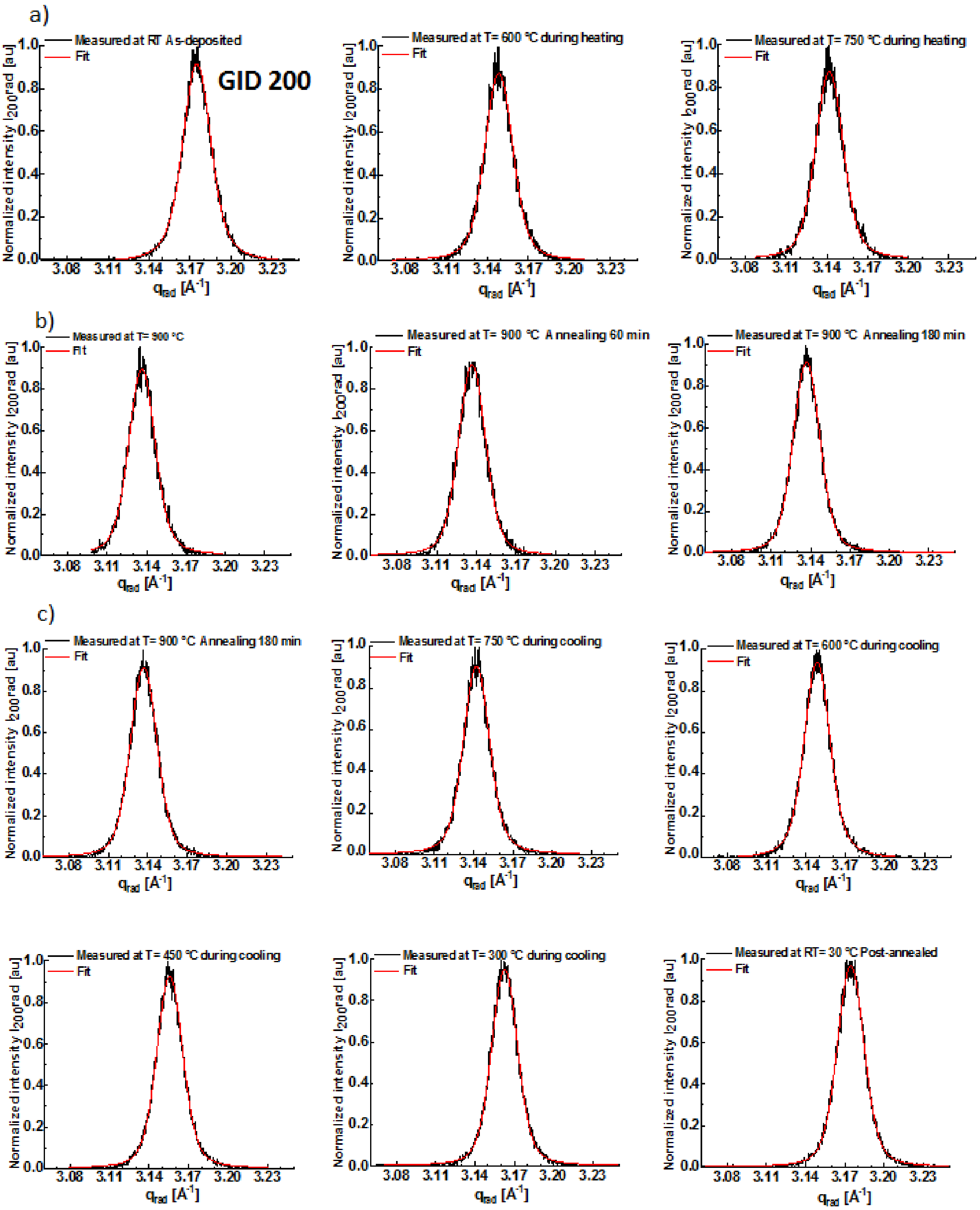
a)
GID 200
Measured at RT As-deposited
Measured at T= 600 °C during heating
Measured at T= 750 °C during heating
b)
Measured at T= 900 °C
Measured at T= 900 °C Annealing 60 min
Measured at T= 900 °C Annealing 180 min
c)
Measured at T= 900 °C Annealing 180 min
Measured at T= 750 °C during cooling
Measured at T= 600 °C during cooling
Measured at T= 450 °C during cooling
Measured at T= 300 °C during cooling
Measured at RT= 30 °C Post-annealed
Fit
Normalized intensity I200rad [au]
qrad [A-1]

Supplementary figure (5)

Diffraction radial profiles corresponding to three different temperatures RT, T=600 °C and 750 °C recorded during heating (black) and during cooling (red line) of a) GID200 and b) XRD002.

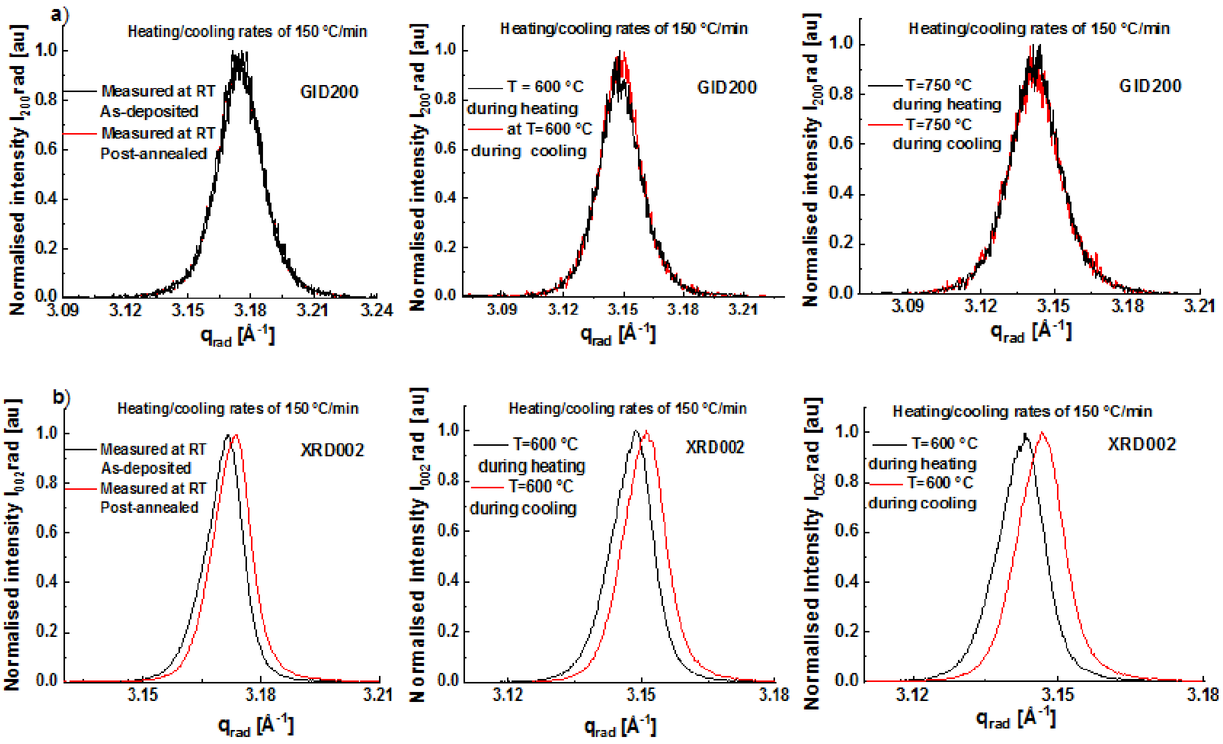

Supplementary tables

Table (1a)*: Structural parameters derived from 2D-RSM measured in in situ during the heating step [A-B] resp. [A'-B']*

	Structural parameters derived from 2D-RSM measured in in situ during the heating step [A-B], [A'-B'] using the fitting procedure of XRD002 reflection												
1	2	3	4	5	6	7	8	9	10	11	12	13	14
	BSTOres peak position XRD002	*Area BSTOres peak*	*BSTO1 peak position XRD002*	*FWHM BSTO1 peak radial*	*Area BSTO1 peak*	*BSTO2 peak position XRD002*	*FWHM BSTO2 peak radial*	*Area BSTO2 peak*	*proportion BSTOres phase*	*proportion BSTO1 phase*	*proportion BSTO2 phase*	*out-of-plane lattice parameter BSTO1*	*out-of-plane lattice parameter BSTO2*
T [°C]	*Qcres* [$Å^{-1}$]	*Ares*	*Qc1* [$Å^{-1}$]	$FWHM1_{rad}$ [$Å^{-1}$]	*A1*	*Qc2* [$Å^{-1}$]	$FWHM2_{rad}$ [$Å^{-1}$]	*A2*	$X_{BSTOres}$[%]	X_{BSTO1}[%]	X_{BSTO2}[%]	*c1[Å]*	*c2 [Å]*
method	*Fit Voigt*	*Fit Voigt*	*Fit Voigt*	*Fit Voigt*	*Fit Voigt*	*Fit Voigt*	*Fit Voigt*	*Fit Voigt*	*Ares /(A1+A2+Ares))*	*A1 /(A1+A2+Ares)*	*A2 /(A1+A2+Ares)*	*2*2π/Qc1*	*2*2π/Qc2*
30	3.16552 ± 0.00114	0.00146 ± 0.00010	3.16878 ± 0.00007	0.01401 ± 0.00005	0.00420 ± 0.00012	3.17227 ± 0.00001	0.00282 ± 0.00076	0.00687 ± 0.00014	11.65 ± 0.58	33.52 ± 0.38	54.82 ± 0.56	3.96568 ± 0.00008	3.96132 ± 0.00002
600	3.14362 ± 0.00022	0.00052 ± 0.00012	3.14634 ± 0.00009	0.01370 ± 0.00005	0.00608 ± 0.00025	3.14926 ± 0.00005	0.00753 ± 0.00004	0.00598 ± 0.00014	4.16 ± 1.08	48.32 ± 2.07	47.52 ± 2.03	3.99396 ± 0.00001	3.99026 ± 0.00006
750	No	No	3.13190± 0.00004	0.01252 ± 0.00002	0.00844± 0.00006	3.14385± 0.00004	0.00767± 0.00002	0.00441± 0.00008	0	65.68± 0.70	34.32± 0.70	4.00051± 0.00005	3.99713± 0.00005

Structural parameters derived from 2D-RSM measured in in situ during the heating step [A-B] resp. [A'-B'] using the fitting procedure of GID200 reflection		
	15	16
	BSOT1 BSOT2 peak position<br><br>*GID200*	*in-plane lattice parameter*<br><br>*BSTO1,2 GID200*
	Qa [Å^{-1}]	*a [Å]*
	Fit Voigt	*2*2π/Qa*
30	3.17494 ± 0.00002	3.95799 ± 0.00002
150	3.16871 ± 0.00002	3.96577 ± 0.00002
300	3.15907 ± 0.00002	3.97787 ± 0.00002
450	3.15469 ± 0.00002	3.98339± 0.00002
600	3.14820 ± 0.00003	3.99158 ± 0.00003
750	3.1418 ± 0.00003	3.99963 ± 0.00004

Table (1b)*: Structural parameters derived from 2D-RSM measured during the in situ during annealing step [B-C] resp. [B'-C']*

	Structural parameters derived from 2D-RSM measured during the in situ during annealing step [B-C], [B'-C'] using the fitting procedure of XRD002 reflection												
1	2	3	4	5	6	7	8	9	10	11	12	13	14
	BSTOres peak position XRD002	*Area BSTOres peak*	*BSTO1 peak position XRD002*	*FWHM BSTO1 peak radial*	*Area BSTO1 peak*	*BSTO2 peak position XRD002*	*FWHM BSTO2 peak radial*	*Area BSTO2 peak*	*proportion BSTOres phase*	*proportion BSTO1 phase*	*proportion BSTO2 phase*	*out-of-plane lattice parameter BSTO1*	*out-of-plane lattice parameter BSTO2*
T [°C]	*Qcres* $[Å^{-1}]$	*Ares*	*Qc1* $[Å^{-1}]$	$FWHM1_{rad}$ $[Å^{-1}]$	*A1*	*Qc2* $[Å^{-1}]$	$FWHM2_{rad}$ $[Å^{-1}]$	*A2*	$X_{BSTOres}$ [%]	X_{BSTO1} [%]	X_{BSTO2} [%]	*c1* [Å]	*c2* [Å]
method	*Fit Voigt*	*Fit Voigt*	*Fit Voigt*	*Fit Voigt*	*Fit Voigt*	*Fit Voigt*	*Fit Voigt*	*Fit Voigt*	*Ares /(A1+A2))*	*A1 /(A1+A2))*	*A2 /(A1+A2)*	*2*2π/Qc1*	*2*2π/Qc2*
900	No	No	3.13664 ± 0.00003	0.01223 ± 0.00002	0.0090 ± 0.00006	3.14006 ± 0.00003	0.00827 ± 0.00003	0.00480 ± 0.00002	0	65.22 ± 0.91	34.78 ± 0.91	4.00632 ± 0.00004	4.00195 ± 0.00004
900+ 60 min	No	No	3.1380 ± 0.00003	0.01192± 0.00002	0.0080 ± 0.00006	3.14122 ± 0.00003	0.00878 ± 0.00003	0.00576 ± 0.00008	0	58.26 ± 0.50	41.74 ± 0.50	4.0045 ± 0.00004	4.00047 ± 0.00004
900+ 180 min	No	No	3.13950 ± 0.00002	0.01154± 0.00002	0.0075 ± 0.00005	3.14203 ± 0.00002	0.00941 ± 0.00003	0.00607 ± 0.00006	0	55.30 ± 0.40	44.70 ± 0.40	4.00262 ± 0.00003	3.99944 ± 0.00003

	Structural parameters derived from 2D-RSM measured in in situ during annealing step [B-C] resp.[B'-C'] using the fitting procedure of GID200 reflection	
	15	16
	BSTO1 BSTO2 peak position<br><br>*GID200*	*in-plane lattice parameter*<br><br>*BSTO1,2 GID200*
	Qa [Å^{-1}]	*a [Å]*
	Fit Voigt	*2*2π/Qa*
900	3.13650 ± 0.00003	4.00646 ± 0.00004
900+ 60 min	3.13670 ± 0.00003	4.00623 ± 0.00004
900+ 180 min	3.13680 ± 0.00002	4.0060 ± 0.00003

Table (1c): *Structural parameters derived from 2D-RSM measured in in situ during cooling step [C-D] resp. [C’-D’]*

	Structural parameters derived from 2D-RSM measured in in situ during cooling step [C-D] resp.[C’-D’] using the fitting procedure of XRD002 reflection												
1	2	3	4	5	6	7	8	9	10	11	12	13	14
	BSTOres peak position XRD002	*Area BSTOres peak*	*BSOT1 peak position XRD002*	*FWHM BSTO1 peak radial*	*Area BSTO1 peak*	*BSTO2 peak position XRD002*	*FWHM BSTO2 peak radial*	*Area BSTO2 peak*	*proportion BSTOres phase*	*proportion BSTO1 phase*	*proportion BSTO2 phase*	*out-of-plane lattice parameter BSTO1*	*out-of-plane lattice parameter BSTO2*
T [°C]	*Qcres* $[\AA^{-1}]$	*Ares*	*Qc1* $[\AA^{-1}]$	$FWHM1_{rad}$ $[\AA^{-1}]$	*A1*	*Qc2* $[\AA^{-1}]$	$FWHM2_{rad}$ $[\AA^{-1}]$	*A2*	$X_{BSTOres}$ *[%]*	X_{BSTO1} *[%]*	X_{BSTO2} *[%]*	*c1* *[Å]*	*c2* *[Å]*
method	*Fit Voigt*	*Fit Voigt*	*Fit Voigt*	*Fit Voigt*	*Fit Voigt*	*Fit Voigt*	*Fit Voigt*	*Fit Voigt*	*Ares /(A1+A2))*	*A1 /(A1+A2))*	*A2/(A1+A2)*	*2*2π/Qc1*	*2*2π/Qc2*
750	No	No	3.1454 ± 0.00002	0.01125 ± 0.00002	0.0080 ± 0.00004	3.14806 ± 0.00003	0.00904 ± 0.00003	0.00564 ± 0.00005	No	58.65 ± 0.36	41.35 ± 0.36	3.99511 ± 0.00002	3.99178 ± 0.00003
600	No	No	3.14960 ± 0.00003	0.01122 ± 0.00002	0.00781 ± 0.00005	3.15235 ± 0.00003	0.00887 ± 0.00003	0.00592 ± 0.00004	No	56.88 ± 0.44	56.88 ± 0.44	3.98983 ± 0.00002	3.98635 ± 0.00002
450	No	No	3.15578 ± 0.00002	0.01124 ± 0.00001	0.00705 ± 0.00004	3.15828 ± 0.00002	0.00891 ± 0.00002	0.00619 ± 0.00005	No	53.24 ± 0.31	46.75 ± 0.31	3.98202 ± 0.00002	3.97887 ± 0.00002
300	No	No	3.16123 ± 0.00002	0.01139 ± 0.00002	0.00753 ± 0.00004	3.16386 ± 2.28917 E-5	0.00815 ± 0.00002	0.00568 ± 0.00004	No	57.00 ± 0.30	42.99± 0.30	3.97515 ± 0.00002	3.97185 ± 0.00002
30	No	No	3.17152 ± 0.00002	0.01187 ± 0.00002	0.00825 ± 0.00004	3.17434± 0.00002	0.00784± 0.00002	0.00508± 0.00005	No	61.89± 0.36	38.11± 0.36	3.96225± 0.00003	3.95873± 0.00004

	Structural parameters derived from 2D-RSM measured in in situ during annealing step [C-D] resp.[C'-D'] using the fitting procedure of GID200 reflection	
	15	16
	BSOT1 BSOT2 peak position GID200	*in-plane lattice parameter BSTO1,2 GID200*
	Qa [$Å^{-1}$]	*a [Å]*
	Fit Voigt	*2*2π/QaGID*
750	3.14216 ± 0.00002	3.99928 ± 0.00003
600	3.14836 ± 0.00002	3.99144 ± 0.00002
450	3.1556 ± 0.00002	3.98216 ± 0.00001
300	3.1626 ± 0.00002	3.97342± 0.00001
30	3.1745 ± 0.00002	3.95855 ±0.00002

Table (2): comparison of the structural parameters between the as-deposited and the post-annealed states

	1	2	3	4
		As-deposited initial state (A,A')	*Post-annealed final state (D,D')*	*changes upon post-annealing*
1	*in-plane lattice parameter a [Å]*	3.95799 ± 0.00002	3.95855 ± 0.00002	*a* ↑
2	*out-of-plane lattice parameter c1[Å]*	3.96568 ± 0.00001	3.96225 ± 0.00003	*c1* ↓
3	*out-of-plane lattice parameter c2 [Å]*	3.96132 ± 0.00001	3.95873 ± 0.00004	*c2* ↓
4	*tetragonal distortion ratio D1 (BSTO1)*	0.99806 ± 0.00003	0.99906 ± 0.00002	*D1 (BSTO1)* ↑
5	*tetragonal distortion ratio D2 (BSTO2)*	0.99916 ± 0.00001	0.99995 ± 0.00001	*D2 (BSTO2)* ↑
6	*proportion BSTO1 phase* X_{BSTO1} *[%]*	33.52 ± 0.38	61.89 ± 0.36	X_{BSTO1} ↑
7	*proportion BSTO2 phase* X_{BSTO2} *[%]*	54.83 ± 0.57	38.11 ± 0.36	X_{BSTO2} ↓
8	*in-plane strain BSTO1* $\varepsilon_{//}$ *(BSTO1)*	-0.10236 ± 0.00149	-0.04934 ±0.00067	$\varepsilon_{//}$ *(BSTO1)* ↓
9	*in-plane strain BSTO2* $\varepsilon_{//}$ *(BSTO2)*	-0.04434 ± 0.00056	-0.00249 ± 0.00077	$\varepsilon_{//}$ *(BSTO2)* ↓

[1] Wojdyr, M. Fityk: a general-purpose peak fitting program. *J. Appl. Cryst*. **43,** 1126 -1128 (2010).

[2] Anthony,M. T. & Seah, M. P. XPS: Energy Calibration of Electron Spectroimeters 2-Results of an Interlaboratory Comparison. *Surf. Interface Anal*., **6,** 107-115 (1984).

[3] Moulder, J. F. Stickle, F. W. Sobol, P. E. & Bomben, K. D. Handbook of X-ray Photoelectron Spectroscopy. *J. Ed: Perkin Elmer Corporation: Eden Prairie*, MN, 1992.

[4] Baniecki, J. D. Ishii, M. Shioga, T. & Miyahara, K. K. Surface core-level shifts of strontium observed in photoemission of barium strontium titanate thin films. *Appl. Phys. Lett*. **89,** 162908 (2006).

[5] Rodrigues, A., Bauer, S. & Baumbach, T. Effect of post-annealing on the chemical state and crystalline structure of PLD $Ba_{0.5}Sr_{0.5}TiO_3$ films analyzed by combined synchrotron X-ray diffraction and X-ray photoelectron spectroscopy. *Ceram. Int.* 2018.
